# Mobile App to Help People With Chronic Illness Reflect on Their Strengths: Formative Evaluation and Usability Testing

**DOI:** 10.2196/16831

**Published:** 2020-03-04

**Authors:** Olöf Birna Kristjansdottir, Elin Børøsund, Marianne Westeng, Cornelia Ruland, Una Stenberg, Heidi A Zangi, Kurt Stange, Jelena Mirkovic

**Affiliations:** 1 Department for Digital Health Research Division of Medicine Oslo University Hospital Oslo Norway; 2 Norwegian National Advisory Unit on Learning and Mastery in Health Oslo University Hospital Oslo Norway; 3 Norwegian National Advisory Unit on Rehabilitation in Rheumatology Diakonhjemmet Hospital Oslo Norway; 4 The Center for Community Health Integration Case Western Reserve University Cleveland, OH United States

**Keywords:** mobile app, self-management, strengths, chronic illness, rheumatology, usability, formative evaluation

## Abstract

**Background:**

Supporting patient engagement and empowerment is increasingly seen as essential in providing person-centered health care to people with chronic illness. Mobile apps helping patients reflect on their concerns as preparation for consultations with their health care providers can have beneficial effects on the consultation quality. However, apps focusing on empowerment and personal strengths are still scarce.

**Objective:**

This study aimed to (1) develop a mobile app to support patients with rheumatic diseases in reflecting on their strengths in preparation for consultations with health care providers and (2) explore patients’ perceived usability of the app in a nonclinical test setting.

**Methods:**

A prototype app was developed based on input from patients and health care providers, as reported in previous studies. The app was designed for use in self-management support settings aiming to promote awareness of strengths and to focus attention on strengths in the patient-health care provider dialogue. The features included in the prototype were as follows: (1) introduction to the topic of strengths, (2) list of examples of strengths to promote reflection and registration of own strengths, (3) summary of registered strengths, (4) value-based goal setting, (5) linking of strengths to goals, (6) summary of all registrations, and (7) options to share summary digitally or as a print version. In this study, the app was refined through a formative evaluation with patients and health care providers recruited from a specialized rheumatology hospital unit. Patients’ perceptions of the app’s usability were explored in a test setting with self-report measurements and semistructured interviews. The interviews were audiotaped, transcribed, and analyzed with directed content analysis. Data from questionnaires were analyzed with descriptive statistics.

**Results:**

Developmental and formative evaluation included 18 patients and 7 health care providers. The evaluation resulted in minor adjustments to the prototype but no major changes in features. The usability testing included 12 patients. All participants found the usability acceptable; the median score on the System Usability Scale was 86.3 (range 70-100). All reported that it was meaningful and relevant to use the app. Out of 12 participants, 9 (75%) reported becoming more aware of their own strengths by using the app; 1 (8%) disagreed and 2 (17%) provided a neutral response. The results on the goal-related feature were mixed, with half of the patients finding it useful to link strengths to concrete goals. A statistically significant positive change from pre- to postintervention was identified on measures of self-efficacy and negative emotions.

**Conclusions:**

In this formative evaluation of a mobile app to promote patients’ reflections on their strengths, patients perceived the app as meaningful and supporting awareness. The results suggest the usefulness of building in functionality to support use of strengths and goal attainment. Further studies on efficacy and usability in a clinical setting, including health care providers, are needed.

## Introduction

Person-centered health care involves a holistic approach with a focus on patient empowerment and engagement [1,2]. It includes helping people with chronic illness to recognize and cultivate their existing strengths and develop new ones [1,3,4]. Strengths have been described as the repertoire of potential attributes that mobilize positive health behavior and promote health and well-being [5]. Strengths are usually contextual and interwoven with goals, interests, values, and situational factors [6]. People with chronic illness report various strengths, such as knowledge, courage, perseverance, kindness, positive emotions, use of coping strategies, and social support [7-9].

Helping patients to identify and engage with their personal strengths is increasingly common in the domain of psychotherapy (ie, positive psychology), as well as in educational and organizational contexts. Research indicates that focusing on strengths can promote outcomes such as motivation, positive affect, and work performance [6,10,11]. Strengths interventions are in line with the broaden-and-build theory stating the important role of positive emotions in promoting a positive spiral of action toward well-being [12]. Including a strengths-based assessment in health care is suggested to provide a more holistic view of patients [1,13]. Assessing strengths can help the health care provider understand each patient’s reservoir of resources that can be utilized in the management of challenges and to promote well-being [13]. Several scales are available for assessment of strengths and resources [13-16]. However, research on strengths assessments in clinical practice is sparse [17,18]. A recent review on strengths interventions published between 2011 and 2016 included 18 studies, of which only three used clinical samples; most studies were done on samples of students or employed adults. Three types of strengths interventions were identified: (1) interventions that helped participants identify their strengths (eg, by reflecting on their best self) without providing instructions on how to use or develop those strengths, (2) interventions that supported identification and use of strengths (eg, using strengths in a new way), and (3) interventions addressing the impact of patients’ use of strengths on others, regulation of use of strengths, or use of strengths in different contexts. Most studies found positive effects on well-being, and the type of intervention was not found to moderate the effect [17].

Interventions aiming to prepare patients for consultations can lead to more active patient engagement during clinical encounters [19]. For example, digital pre-encounter communication interventions that help patients prepare for a conversation about their symptoms and problems have shown positive effects on communication and consultation quality [20-22]. Following a strengths assessment, the health care provider and the patient can explore together how the patient’s strengths have helped previously and how they might help in the current situation [13]. However, interactive digital interventions that support patients in exploring and reporting their strengths in a clinical setting are generally still few [18].

Two prior studies have explored insights from patients and health care providers regarding the use of a mobile app aiming to support reflection and dialogue about patients’ personal strengths [23,24]. People with different chronic illnesses were generally positive toward using technology to help identify and discuss their personal strengths in clinical consultations. Patients suggested the app should include examples of strengths reported by other patients with chronic conditions and an option to extend the list with personal items using intuitive and engaging user-interface design [23]. Health care providers working in a rheumatology setting described how they supported their patients in mobilizing their strengths and emphasized the importance of communication skills, exploration of values, and goals to mobilize strengths, as well as patient education [24]. The health care providers were also generally positive toward the idea of an app to support the patients’ reflections on strengths as a preparation for consultations to promote self-management [24].

The aim of this study was to (1) develop a mobile app to support reflection and dialogue about personal strengths among patients receiving self-management support and (2) explore how patients perceived the app’s usability, as well as its potential effects on emotions and self-efficacy, in a nonclinical test setting.

## Methods

### Overview

**Figure 1:**
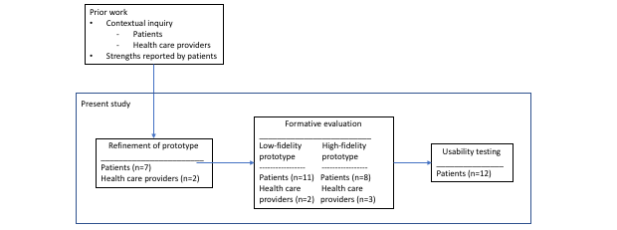
Overview of the development and evaluation process.

This study used a person-centered approach for design and development of the app, with mixed methods for evaluation [25]. The study was approved by the Privacy and Security Protection Committee of a major hospital in Northern Europe. Participants were recruited from two units of a rheumatology specialist department—one of which arranged outpatient self-management programs, whereas the other had an inpatient rehabilitation unit—and from a patient-research partner advisory board. Inclusion criteria for patients were being 18 years of age or older, being fluent in Norwegian, and having a rheumatic disease. The only inclusion criterion for health care providers was to have experience with health care for people with rheumatic diseases; they were recruited from the inpatient rehabilitation unit. All participants provided written informed consent and patient participants received a gift certificate as compensation for time spent and travel expenses. Information about patients who were invited to participate but declined was not registered. Figure 1 gives an overview of the development and evaluation process.

### Development

#### Refinement of the Prototype

Design of the prototype was guided by suggestions from people with chronic illness who had participated in designing a preliminary low-fidelity (ie, paper) prototype [23] as well as feedback on this prototype from health care providers [24]. The preliminary prototype offered the following main features: (1) a preliminary list of strengths with *yes* and *no* response options, (2) registration of a health-related goal, (3) marking of 3-5 most relevant strengths, (4) linking strengths and goals, and (5) an easy way of sharing results with a health care provider.

In a prior study, people with chronic illnesses were asked about their strengths [7]. This resulted in a preliminary list of strengths items that was used in a study exploring patients’ requirements for a strengths-based preconsultation app [23]. The list of strengths items from this previous work was further refined (eg, number of items reduced and wording clarified) in this study during discussions between authors and based on feedback during formative evaluation. The items on the list were meant to serve as examples of strengths to support reflection rather than to be a formal assessment.

The development process involved a multidisciplinary team from a hospital research center comprising behavioral scientists, an information technology scientist and developers, a designer, specialists in the development of content and functionality in eHealth apps, and user representatives—two women with rheumatic disease. The intention was to make an app available without cost to other users once its efficacy had been demonstrated and guidelines constructed to facilitate implementation of the app into existing health care contexts. The hospital’s Privacy and Security Protection Committee was consulted for approval of privacy and security requirements of the app.

To refine the features of the prototype, patient representatives with rheumatic diseases were invited to participate in a workshop. They were members of the patient advisory board at the hospital where recruitment took place. The workshop was led by a team member who is a specialist in eHealth app development. The workshop included a short introduction, individual reflection, and discussion. Participants were asked to identify strengths from a predefined list, write about their values and goals, and to reflect on how the strengths could be linked to their goals and values. Input from the workshop was audiotaped and summarized by two team members (OBK and MW). Feedback on main features of the prototype and context of use was gathered in one workshop with health care providers from the inpatient unit. The workshop was audiotaped and main results summarized by two team members (OBK and MW). As a service design method [26], a simple journey map was made to help visualize where using the app might be appropriate in the context of a rehabilitation program.

#### Formative Evaluation

##### Overview

The formative evaluation included testing of low- and high-fidelity versions of the prototypes. Patients participated in an individual testing session. They were guided by a moderator and asked to think aloud during the process to elicit feedback [26]. Testing sessions with patients were video recorded and sessions with health care providers audiotaped. All patients were asked to complete a background information survey, including questions on age and gender.

Data on usability were extracted from observation notes and video and summarized by two team members (OBK and MW) into the following categories: *functionality*, *content issues*, *navigation*, and *nice to have*. This summary was then discussed within the project group and used as feedback for iteratively adjusting the prototype between testing rounds.

##### Low-Fidelity Prototype Testing

For this phase, participants were recruited from three settings within the hospital. Patients recruited from the self-management programs and the inpatient unit were informed of the study by their health care providers. Those who expressed interest were contacted by a research team member by telephone and provided with more information about the study. Members of the patient advisory board were sent an email with study information and an invitation to participate. The project’s user representatives also participated in this phase of testing.

The first low-fidelity prototype was developed using a combination of paper sketches and app screenshots that simulated the app’s features and flow. Prototyping was done using the POP app by Marvel [27]. It included the following features and functionality: (1) information in text and audio, (2) strengths reflection: list of strengths presented with response options *yes* and *no*, (3) possibility to add notes to strengths, (4) overview of marked strengths, presented within four categories, (5) value-based goal setting, to be entered into a text box, (6) linking of strengths to goals, and (7) sharing, indicated with a printout symbol (see Multimedia Appendix 1).

The testing procedure was piloted with coworkers to ensure feasibility of the procedure before it was used with patients. The testing sessions were held at the research center. The prototype included multiple pieces of paper that were handled according to what the participant “clicked” on to simulate activities on a touch screen. Participants were encouraged to write on the paper to contextualize their interaction with the prototype, for example, if or how they wanted to express or visualize their strengths or adjust the interface. In addition to the participant, there were three facilitators in the room. One person moderated the process, one administered the paper parts of the prototype according to participants’ choices on the screen, and one observed and assisted. The team of facilitators had interdisciplinary backgrounds: researcher, system developer, and content manager. Health care providers participated in a group session where the prototype was shown and discussed, using a journey map to aid discussion on context for use.

##### High-Fidelity Prototype Testing

Based on feedback from the low-fidelity prototype testing, a high-fidelity prototype was programmed using the software program Unity (Unity Technologies) [28]. For this phase, participants were recruited from the inpatient rehabilitation unit only. Patients were informed about the study by a health care provider and for those willing to participate, a testing session was scheduled at the unit. The high-fidelity testing procedure was similar to the low-fidelity prototype testing but with one fewer facilitator in the room. Health care providers at the same unit were also invited to test the prototype and provide feedback; they each tested the prototype with either one or two providers per session. The sessions with health care providers were audiotaped and included a moderator but no facilitators. Lastly, the interface was explored by an external interaction designer who was provided with the intended context of use and then performed an informal heuristic evaluation of the app on his own. He provided feedback on user-friendliness issues and suggestions for improvements in a meeting with team members.

### Testing Usability of the Final Version of the Mobile App

Participants were recruited from self-management programs for people with a rheumatic disease. The program facilitator presented the researcher, research assistant, and/or patient representatives to the group. The study was presented and those who were interested provided their contact information. Subsequently, they were called and given more detailed information about the study, and a date for participation was agreed on. The usability testing took place at the research center.

After the participants had filled out self-report questionnaires, the researcher presented a scenario and the usability tasks. Participants were asked to imagine being invited by their health care provider to use the app to reflect on strengths and goals, before a consultation. The description of the scenario was available in written format during the testing session. During the testing, the participant was alone in the room. The participant was informed that the testing would take approximately 30 minutes and that the researcher would check in after about 20 minutes to see if they needed more time. After the usability testing, the researcher printed out a copy of the strengths report for use in a posttest interview.

### Self-Report Measurements

#### Overview

Prior to the usability test, participants filled out a questionnaire on background information and a study-specific item on strengths. To explore potential usefulness, participants were also asked to fill out outcome measures on emotions and self-efficacy before and after testing the app. Lastly, after the usability test, participants were asked to fill out a study-specific outcome measure on perceived usefulness and a usability scale. Statistics were analyzed using SPSS Statistics for Windows, version 25.0 (IBM Corp). The related-samples Wilcoxon signed-rank test was used to compare the median of differences between pre- and posttest scores.

#### Usability

Participants also completed an 11-item study-specific questionnaire about the functionality and perceived usefulness of the app. Response options ranged from 1 (*strongly agree*) to 5 (*strongly disagree*). See the Results section for an overview of items. Usability of the app was measured with the System Usability Scale [29], a 10-item measure with five response options ranging from *strongly agree* to *strongly disagree*. Scores were converted so that all values were between 0 and 4, with 4 as the most positive response. See the Results section for an overview of these items. The converted scores were summed up and multiplied by 2.5, leading to a value range of 0-100, with 100 being the most positive response. In case of a single missing response, the item was scored with a neutral value (score=2).

#### Emotions and Self-Efficacy

Self-efficacy and emotions were measured to explore whether the testing session would lead to any preliminary indications of changes. Positive and negative affect was measured with the Positive and Negative Affect Schedule [30]. This measure includes subscales for positive (eg, interested and enthusiastic) and negative affect (eg, guilty and scared), with 10 items in each. The participant was asked to indicate to what extent he or she was currently feeling a specific emotion, with the response alternatives *very slightly or not at all*, *a little*, *moderately*, *quite a bit*, and *extremely*. Subscale scores ranged from 10 to 50, with higher positive affect scores representing higher levels of positive affect and lower negative affect scores representing lower levels of negative affect. In case of a single missing item on the preintervention measurement, the item was given the same score as the reported postintervention item.

A revised version of the Arthritis Self-Efficacy Scale was used to measure self-efficacy to cope with pain (five items) and others symptoms (six items) [31,32]. In this scale, the respondent is asked about level of certainty in being able to undertake specific tasks (eg, decrease pain, continue daily activities, control fatigue, and do something to feel better). Five response options are provided from *very uncertain* to *very certain*. A score for the *pain* subscale (range 0-20) and the *other symptoms* subscale (range 0-24) was calculated, with higher scores representing higher levels of self-efficacy. In case of a single missing item on the preintervention measurement, the item was given the same score as the reported postintervention item.

### Posttest Interview

After the participants had filled out the posttest outcome measures, they were interviewed by the first author (OBK). During the interview, the printout of the strengths overview and the app were available and referred to. The interview was semistructured (see Textbox 1), audiotaped, and transcribed. The data were analyzed using content analysis [33] directed by predefined categories following the interview guide as follows: (1) the app as a reflection support, (2) the app as a dialogue support in health care, and (3) user-friendliness and suggestions for improvement. The interviews were intended to supplement the questionnaire data on the experience of using the app.

**Examples of questions from the interview guide.**

How did you experience being asked about your strengths through the app?

How was it to describe your strengths with the app versus without it (as you did on paper before the usability testing)?

What are your thoughts about using the app as preparation before a consultation/conversation with your health care provider? Do you have a health care provider in mind? If so, what profession/setting?

Do you think it would be helpful to use the app, without sharing the report? Please elaborate.

What are your thoughts about the usefulness of the app in relation to a group-based self-management program, such as the one you recently attended?

How easy was it to navigate in the app? Do you have suggestions for improvement?

## Results

### Design and Development: Refinement of Main Features

#### Workshop With Patients

A total of 7 patients participated: 4 out of 7 (57%) were women. The age range was 31-63 years (median 56 years). The patient representatives in the project participated in the workshop and are included in the sample description. Patients highlighted the need to acknowledge the fluctuating nature of the illness and the variation in perceived strengths. Patients wanted to be able to reflect on previous use of strengths. Acknowledging challenges in addition to strengths was considered important. There was some confusion about how to make a link between strengths, values, and goals. Some found goal setting and value reflection easy and motivating, while others found it challenging to find realistic and inspiring goals.

#### Workshops With Health Care Providers

Two health care providers participated in a workshop where the idea of the app was discussed with a focus on potential context of use; this was supported by a journey map developed in preparation for the workshop. They described the importance of taking into consideration the patient’s readiness for change. The app was viewed as potentially useful (1) when exploring strengths of patients in general (eg, what is currently working well) and, more specifically, (2) when working with goal setting and self-efficacy toward goals. The health care providers suggested adding the possibility to register obstacles to reaching goals, as well as overview of and feedback on progress.

### Formative Evaluation

#### Low Fidelity

In the first of three rounds, the prototype was tested by 3 patients (2/3, 67% women; age range 42-63 years, median 57 years), including the patient representatives in the project. In total, 5 women participated (age range 28-64 years, median 50 years) in the second round and 3 women (age range 30-77 years, median 43 years) in the third round. A total of 2 participants had also participated in the previous workshop. Finally, the patient representatives in the project tested the prototype again.

In general, participants appreciated the list of strengths items and all were able to find several or many relevant items. A few changes were made based on feedback during this evaluation phase. Since some patients reported that it was difficult to assign strengths to an *either/or* category, the response alternative *partially* was added. Also, some patients reported appreciating being able to view the list of strengths registered with a *no*. They perceived this as feedback on self-management strategies they could potentially work on or do more of. Other adjustments included simplifying the structure of the overview, including more information and guidance (eg, by adding instructions and examples of goals and pop-up information with encouragement and guidance), and the option to share a selection of strengths with the provider instead of the complete overview.

A few changes that were made and tested did not work well and were, therefore, not implemented in the high-fidelity prototype (eg, the option to choose to start with goal setting rather than selecting strengths and the option to mark strengths items the user wanted to use more actively in the future). In addition, participants made several suggestions for added functionality that were not implemented due to limited resources. Examples include functionality related to goals (eg, progress bar and reminders), reminders of their strengths, registration of strengths uses, tailored information on how to use their strengths, registration of challenges, and a forum for sharing between users.

Two health care providers gave their feedback on the prototype in one shared session. They were generally positive toward the functionality. They emphasized the importance of the possibility of adding one’s own formulations and using the notes function to write about how the strengths could help them toward a goal. They reported that the items, categories of strengths, and response alternatives seemed appropriate.

I absolutely think this is a way to activate the patient in the process, a good tool.

Health care provider

The guidance and examples of goals were appreciated, but several participants commented that the instructions on writing a goal needed clarification. They suggested that the app could be introduced by the patient’s primary nurse in the context of conversations about goal setting and achievement.

#### High Fidelity

The high-fidelity prototype was a mobile app called Styrkefunn in Norwegian (meaning *strengths discovery*). To introduce the app and the concept of strengths, a short animated video was made and included in the app. The video included a metaphor of being out at sea, representing illness, and needing to take a second look at the options, representing strengths, available to keep the boat going forward in the right direction. A few adjustments were made on the app based on feedback from low-fidelity prototype testing; for example, options were added to allow users to mark strengths as *not relevant* (ie, to skip them), to add subgoals, and to link strengths to both levels of goals. The decision to not prioritize a digital sharing functionality (eg, by direct integration to electronic health records) was taken, due to issues related to privacy protection regulations and project resources. For the purpose of the usability testing, this functionality was implemented by enabling a feature that allowed for making a printout of the overview of all registrations made in the app. The list of strengths was refined based on feedback from participants (eg, some items were merged or divided and the language edited). The high-fidelity prototype included 42 strengths items categorized into four domains: (1) qualities (eg, I am persistent and I am creative), (2) strategies (eg, I seek the knowledge I need, I have a healthy lifestyle, and I take care of myself), (3) external resources (eg, I have someone who understands me and I have health care providers whom I trust), and (4) joy and meaning (eg, I prioritize that which is important to me and I have activities that I look forward to) (see Multimedia Appendix 2 for the refined list).

The high-fidelity prototype was tested in two rounds, with 3 patients participating in each (5/6, 83% women; age range 34-50 years, median 45 years). Additionally, the 2 patient representatives within the project and 3 health care providers from the inpatient unit participated; 1 health care provider had participated previously. The feedback from the patients indicated that the strengths reflection worked well, both the items and the response alternatives. All appreciated the introductory video. Several user-friendliness issues were experienced, indicating a need for better navigation and guidance in the goal part of the app. The three health care providers were positive toward the app; they reported liking the introductory video and the list of strengths items.

I think this can be a very nice tool to use ... If we ask the patients about their resources, then they are unsure about how to start answering, I don’t think they understand the question ... but if you get the suggestions, then it is easier ...

Health care provider

The health care providers suggested that the goal part needed more instructions and ideally the app should be designed to support self-management beyond the reflection and consultation.

**Figure 2:**
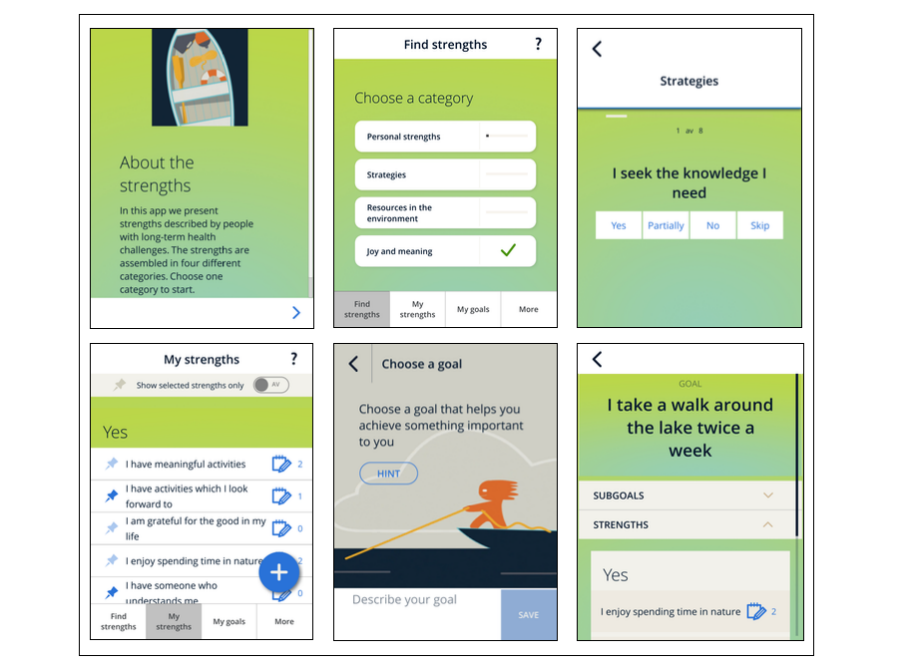
Screenshots of the app.

Finally, patients, health care providers, and the interaction designer experienced some usability issues related to navigation (eg, pop-up guidance was found confusing and it was unclear where to access strengths and subgoals from the goal page). The designer also pointed out synchronicity issues in layout. The issues were categorized as mainly cosmetic and minor and were addressed by the project team, but more functionality was not added due to limitations in project resources. Figure 2 shows screenshots of the app’s interface after refinement from formative evaluation.

### Usability Testing of the Digital App

#### Participants

Participants (N=12) were mainly female (11/12, 92%) and had different (ie, one or more) rheumatologic diagnoses (eg, psoriasis arthritis, fibromyalgia, spondyloarthritis, and rheumatoid arthritis). Median age was 50.0 years (range 30-76). Out of 11 participants, 9 (75%) reported a high degree of experience with a mobile device and 3 (25%) reported having some experience. See Table 1 for additional descriptive information.

**Table 1:** Participants’ demographics and duration of pain.

Variable				Participants (N=12), n (%)^a^	
Married or cohabiting				9 (75)	
**Employment**					
	Full-time work				3 (25)
	Part-time work				2 (17)
	Sick leave				2 (17)
	Disability benefits				5 (42)
**Education**					
		Elementary or upper secondary school		4 (33)	
		Education beyond upper secondary school		8 (67)	
**Pain duration (years)**					
			<1	0 (0)	
			1-2	4 (33)	
			2-4	1 (8)	
			4-6	0 (0)	
			6-10	3 (25)	
			>10	4 (33)	

^a^Due to rounding, percentages may not add up to 100%.

#### Usability

On the study-specific question about strengths, prior to using the app, participants listed between 3 and 10 strengths (eg, family and friends, being able to work part time, creativity, positive mindset, being mentally strong, being goal oriented, regular exercise, healthy diet, insight into what nurtures and what does not, valuable work, pets, and support from health care providers). When using the app, all participants registered strengths. The number of strengths registered in the *yes* category ranged from 6 to 35 (median 25), the number of strengths in the *partially* category ranged from 6 to 26 (median 17), and the number of strengths categorized as *no* ranged from 0 to 7 (median 1). Out of 12 participants, 11 (92%) registered a goal and 7 (58%) also registered a subgoal; 8 participants (67%) linked their strengths to a goal.

All participants reported finding it meaningful and relevant to use the app. Out of 12 participants, 9 (75%) reported becoming more aware of their strengths by using the app and 10 (83%) found it useful to see the overview of strengths. The results on the perceived usefulness of registering goals were mixed: 5 out of 12 participants (42%) found it useful, whereas 4 (33%) were neutral in their opinion, and 2 (17%) did not find it useful. Out of 12 participants, 6 (50%) found it useful to link strengths to a goal but 5 (42%) provided a neutral response. See Table 2 for more detailed results from the study-specific questionnaire.

The median score on the System Usability Scale was 86.3. One missing score was replaced with a neutral score. The scores ranged from 70 to 100 (ie, all participants found the usability acceptable). Results from half of the participants met criteria for excellent usability (ie, score >85.5) [34]. The statements in the usability questionnaire alternated between expressing positive and negative opinions. Disagreement with a negatively formulated statement was scored as a positive response. One item—*I think that I would like to use this system frequently*—received two negative responses and five neutral responses. Another item—*I found the system unnecessarily complex*—received one negative response. Other items received neutral responses and mostly positive responses (see Table 3 for details).

**Table 2:** Perceived usefulness of the app among patients (N=12).

Usefulness statement	Response, n			
	Disagree or strongly disagree	Neutral	Agree or strongly agree	Missing
It was meaningful and relevant to use the app	0	0	12	0
I became more aware of my own strengths by using the app	1	2	9	0
It was useful for me to see an overview of my own strengths	0	2	10	0
It was useful for me to be able to register a goal	2	4	5	1
It was useful for me to be able to link the strengths to concrete goals	0	5	6	1
I liked the possibility to register my own notes	0	4	7	1
For me it would be relevant to share the overview of my strengths with my health care provider	0	4	8	0
It was boring to use the app	9	1	2	0
It was demanding for me to use the app	11	0	1	0
I would like to have the app on my own mobile device	2	3	7	0
I would recommend the app to others in my situation	0	3	9	0

**Table 3:** Questions and results from the System Usability Scale (N=12).

Usability statement	Response, n			
	Negative^a^	Neutral	Positive^b^	Missing
I think that I would like to use this system frequently	2	5	4	1
I found the system unnecessarily complex	1	1	10	0
I thought the system was easy to use	0	1	11	0
I think that I would need the support of a technical person to be able to use this system	0	0	12	0
I found the various functions in this system were well integrated	0	1	11	0
I thought there was too much inconsistency in this system	0	1	11	0
I would imagine that most people would learn to use this system very quickly	0	1	11	0
I found the system cumbersome to use	0	0	12	0
I felt confident using the system	0	2	10	0
I needed to learn a lot of things before I could get going with this system	0	0	12	0

^a^*Agree* or *strongly agree* for negatively formulated items; *disagree* or *strongly disagree* for positively formulated items.

^b^*Agree* or *strongly agree* for positively formulated items; *disagree* or *strongly disagree* for negatively formulated items.

#### Self-Efficacy

A statistically significant positive change from preintervention to postintervention was identified on both the *pain* and the *other symptoms* subscales. The median preintervention score for the *pain* subscale was 12.0 (range 5-16) and the median postintervention score was 14.0 (range 6-17), resulting in a median difference of 2.0 (*P*=.01). The median preintervention score for the *other symptoms* subscale was 16.0 (range 8-22) and the median postintervention score was 17.0 (range 10-22), resulting in a median difference of 1.0 (*P*=.03). One participant did not fill out this questionnaire and one missing item was replaced.

#### Emotions

There was no improvement postintervention on the positive emotion subscale (*P*=.77) (median preintervention score 34.0, range 31-46; median postintervention score 35.0, range 28-45). A statistically improved postintervention score was identified on the negative emotion subscale (*P*=.02) (median preintervention score 12.0, range 10-23; median postintervention score 11.0, range 10-16). One participant did not fill out this questionnaire and three missing items were replaced.

### Qualitative Results

#### The App as a Reflection Support

Out of 12 participants, 8 (67%) described positive experiences related to reflection on strengths after using the app. They described feeling more aware and reminded of their strengths and how that felt good. They described how the app, in particular the strengths items, made it easier to reflect on strengths than when they reflected without the app, as they were asked to do in the pretest questionnaire. The overview of strengths and goals at the end was appreciated and a few mentioned liking the option to print it out to keep visible as a reminder and for a continued awareness process.

You go in, and then you talk about everything that’s wrong, and then you get some medicines. And then you hope it will work out OK, sort of. While on the other hand, thinking about these strengths, just becoming a bit aware of how good your situation really is, even if your body isn’t a hundred per cent. Getting a bigger picture of things, I think this is really useful.

Participant #1

Out of 12 participants, 5 (42%) described already being generally aware of their strengths. Some of them had experience of a strengths-based approach in their work or education in leadership or health care, and 3 (25%) described the reflection on strengths as a neutral experience, attributing this to the fact that it was not new to them.

I think I’m very aware of my strengths. I’ve worked a bit with this before, so ... and I am also very positive by nature. So for me, I think I have such a clear picture of this at the moment, that I felt that with the short time I used the tool, it was maybe not so useful for me.

Participant #11

The three response alternatives for grading of strengths were generally well received. Some reported finding the items they had marked as *partially* as the most important ones to discuss with the provider and also as something to work on themselves. Some described liking the inclusion of the *no* items on the overview, as this was found to reflect areas for improvement. One participant described that it was difficult to choose response alternatives since strengths varied over time, and another described finding it difficult grading the strengths without having a specific context in mind. Two participants found the strengths items somewhat vague or general. One described missing an item on well-being where she could specify how she created moments of well-being during the day. A couple suggested adding items on negative traits so that you could also reflect on what negative traits were not a description of you.

The participants had different experiences related to the goal setting part of the app. About half of the participants appreciated this functionality and said that they had written a goal of their own without difficulty.

That was maybe the one that I had most benefit from. Yes, because I have a very clear goal. All along I’ve had in my mind that I would go back to work. This is very important to me. But when I had to set up subgoals, I became much more aware that, okay, there was actually a strong connection here. These are things that you must be able to get done so that you will manage to reach that goal, in a way. So that process of becoming aware, and, and so which of my strengths I can use to achieve that. That is what I gained the most from.

Participant #7

Others described setting a goal as difficult and, similarly, some described linking strengths to goals as challenging. A few expected a clearer link or transition from registering strengths to setting goals. Some perceived the written guidance available in the app as helpful and adequate for goal setting, but not all had seen this information and some stated they needed more guidance to be able to set a goal in a constructive way. A few had goals they felt unsuccessful at reaching, causing them to feel bad about themselves. Some said they needed a clearer picture of the setting and what type of health care personnel would be providing follow-up to know what kind of goal to set. Some participants described expecting the app to make goal suggestions based on their registration of strengths (eg, suggest a goal related to building or using more strengths for items marked as *partially*).

#### The App as a Dialogue Support in Health Care

The majority of the participants were generally positive toward sharing the overview of strengths and goals with their health care providers. However, several participants reported concerns about the use of the overview in a dialogue with a health care provider. Some wondered if the health care providers were able to use the overview in a useful way and if they had the time available to do so in settings where learning and mastery were not prioritized topics. A couple of participants expressed a concern that giving the health care provider an overview of the patient’s strengths might lead to less attention being paid to the need for help. A few suggested that the strengths registered as *not relevant* or *partly relevant* were those that might be most relevant to discuss with the health care provider. When asked about which of their health care providers might be relevant for the dialogue on strengths, many of the participants said that it would not be their general practitioner or rheumatology specialist, mostly due to time limitations, but also because of not meeting the same physician at follow-ups. Several other health care professionals were suggested as being more suitable for a conversation that included the strengths summary (eg, psychomotor therapists, psychologists, social workers, nurses, and psychiatric nurses). Almost all said they believed the app could be useful in a self-management program setting, either as a reflection to prepare them for the program, as a reflection exercise to prepare for group discussions, or as a follow-up and reminder after the program if more self-management support functionality was included.

Several described how the topic of strengths was neglected due to a focus on treatment and time limitations, and how they had to search for the health care provider that could help them work toward better self-management of the illness beyond medical treatment. Conversely, a few others described having received health care that addressed their support needs in a positive way.

No, there is just no focus on that. There just isn’t. It’s all about swollen joints and where you have pain. Inflammation and medication. That’s what it’s about. It even took many years. After all, I’ve been ill for a long time. So it took many years before I heard about the offer of courses and training in life skills. And ... yes, things like that.

Participant #3

#### User-Friendliness and Suggestions for Improvement

The majority of the participants found the app generally easy to use. Still, half of the participants reported some minor usability issues (eg, being unsure if something was saved before continuing to the next page or how to move back a step, finding the buttons too sensitive or quick, and missing a better overview of the functionality from the start). A few did not notice the possibility or understand how to add subgoals to the main goals or to link their strengths to a goal (eg, did not see where to press to find the text field for subgoals). Many of the participants mentioned liking the animated instruction video but a few also suggested adding more information, specifically about the health care provider receiving the overview and what to expect from the follow-up dialogue. Participants provided several suggestions for added functionality, and some had expected an app with more functionality relating to self-management support. The most common suggestions involved the following:

1. The app should give advice and suggestions based on the strengths registration about how to improve and/or build strengths and a feature to help users leverage their strengths (eg, providing a progress overview of strengths use or help with setting a strength-related goal).
2. Refining the goal-setting and goal follow-up features within the app (eg, more guidance in setting goals, reminders, view of progress, and encouragement).

A few also mentioned the possibility to add a feature aiming to help the user prepare for a consultation (eg, what to request help for and how).

## Discussion

### Principal Findings

In this study, we developed a mobile app to support reflection on personal strengths for patients in need of self-management support due to a chronic illness and then tested its usability. In general, the patients reported that the app supported reflection on their strengths and was easy to use. The results on the goal-setting part of the app were mixed, as many of the patients did not find it helpful to register a goal and/or reflect on which of their strengths would help them toward achieving the goal. Even though the main prototype features were based on prior work and were not changed in any major ways during the formative evaluation, both patients and health care providers gave feedback that resulted in adjustments of the design of the prototype. Minor functionality changes were made (eg, to enable patients to mark strengths as partially possessed and to skip items considered irrelevant, added guidance on goal setting, and improved navigation). The need for only small adjustments might be due to considerable prior work during the inquiry phase [23,24]; however, testing in clinical practice might have resulted in more adjustments being made (eg, on the goal-setting part).

During the formative process, patients reported finding it important to include in their overview of strengths not only the strengths they identified with, but also the ones they identified with partially or not at all. Patients described how these items, particularly items marked *partially*, were areas they wanted to work on and focus on in a following consultation. This is in line with a view of strengths as malleable qualities that can be cultivated, as opposed to fixed traits [6]. The inclusion of self-management behaviors on the list of strengths, and not solely character strengths or individual qualities, can also contribute to explaining the importance of not only identifying strengths currently present, but also including strengths that have been used previously or that the user wants to develop. Skills and behavior are commonly included in definitions of strengths [18,35]. Overall, the list of strengths was positively received by both patients and health care providers; its inclusion of 42 items was not perceived as overwhelming. Participants, both patients and health care providers, generally agreed that the reflection on strengths was important in itself, and that it promoted positive emotions, although some patients did point out the possibility of some negative effects on the interaction with clinicians. The positive perceptions by patients and health care providers are similar to results reported from related work done at community centers in the United States, to develop an app for patients from disadvantaged backgrounds. However, in that setting, additional video prompts were needed to help patients see and report their strengths [18].

Both patients and health care providers had many suggestions for improving the app by adding functionality that would extend its usefulness beyond the reflection and the subsequent consultation (ie, providing support and guidance on using strengths, cultivating strengths, and goal achievement). Therefore, a potential next iteration of the app could include more educational content and exercises. Since no general theoretical model for strengths-based assessments or interventions is available to date [35], the added material should be anchored in evidence-based behavioral change theory supporting a strengths-based approach, such as acceptance and commitment therapy [36] or goal-setting theory [37]. Many, but not all, participants agreed on the importance of viewing the strengths in relation to a goal. However, the results indicate clearly that the goal part of the app needs to be expanded to include more guidance on goal setting and additional functionality to support goal achievement over time.

The results indicated changes in a positive direction in self-efficacy and negative emotions. This is promising, but due to the small sample and study design, these results need to be interpreted with caution; more research is needed to explore whether the app can promote clinically significant changes. Interestingly, the results did not show changes in positive emotions and were, therefore, not in line with models and evidence suggesting positive emotions as a mediating factor between positive activities and well-being [12,17].

Helping patients to recognize and cultivate their strengths relevant for their individual values is essential for person-centered health care [1]. However, a cautious approach toward interventions promoting standardized ways or tasks to cultivate strengths is warranted, since different kinds of interaction and support will be needed depending on the person and context [1]. Health care providers’ beliefs in the importance of providing person-centered care, their contextual possibilities, and communication skills are crucial [1,38]. A strengths-based app as presented here might potentially be a helpful supplement for health care providers in engaging their patients. More research on the efficacy of this app is needed before making it available and recommending it to users. Exploring the use of the app in the context of self-management programs might be a feasible next step. Even though one specific patient group was involved in this study, the app itself was made without any reference to a specific diagnosis and might, therefore, be suitable for people with different chronic illnesses. It might also be of interest to test the use of the app in the context of the patient’s home and involving a relative or a friend, as apps designed to improve communication between patients and health care providers can also prompt patients to share their experiences with relatives [39]. Framing the task of identifying strengths as reflecting on positive memories of self or visualizing best possible selves seems to be promising [40] and might be important to consider when asking patients to identify strengths as a way to cultivate a strengths-based growth mindset.

### Limitations

Limitations of the study include the small sample size with few participants in each iteration, a sample with a majority of women with one specific chronic disease (ie, rheumatic diagnosis), and the recruitment from a specialized hospital unit and patient advisory group. A small number of participants is not uncommon in formative evaluation studies but a larger number would have been preferable to promote variance in feedback. A majority of the patients invited to participate were women; thus, adjustment in recruitment methods might have been necessary to include a more balanced sample. Men and patients recruited from primary care might have experienced the app differently, since they might be expected to have less experience with extended self-management support and programs. During the testing sessions, the participants met with researchers and team members involved in the development of the app. This might have resulted in a bias toward positive results, something that might have been addressed by involving an independent person during the testing. The inclusion of two patient representatives throughout the project was a strength, as they grew confident in giving their honest opinions over time and had the advantage of consulting each other. The development platform for the study was Unity, a platform most suitable for creating games. This turned out to be a suboptimal choice for this project, as the platform was new to the developer team and the development took more resources and time than anticipated; also, no game-like features ended up being included in the app. During the formative evaluation task, task success was evaluated by observation; in the usability test of the app, evaluation took the form of a postintervention interview and assessment of what had been registered in the app. A more systematic use of a usability model might have led to more detailed measures of usability (eg, time on task or error rate) [41]. Research is needed on the usability of the app in a clinical setting and on how its usefulness is perceived by health care providers.

### Conclusions

In this formative evaluation of a mobile app to promote patients’ reflection on their strengths, patients perceived the app as meaningful and supporting awareness. The results provide preliminary evidence for beneficial effects on negative emotions and self-efficacy. The results suggest building in functionality to support strengths use and goal attainment; the results show the utility of a careful process of app refinement with multiple stakeholders. Further studies on efficacy and usability in a clinical setting, including health care providers, are needed.

## Appendix

Multimedia Appendix 1 Screenshots from the low-fidelity prototype (in Norwegian).

Multimedia Appendix 2 Refined list of strengths used in the Styrkefunn app (in Norwegian and English).
