# Supplementary figures and images for: Mobile App to Help People With Chronic Illness Reflect on Their Strengths: Formative Evaluation and Usability Testing
Source: JMIR Form Res. 2020 Mar 4;4(3):e16831. doi: 10.2196/16831 (PMC7081135; doi:10.2196/16831)

Multimedia Appendix 1:

Screen shots from low-fidelity prototype (in Norwegian)


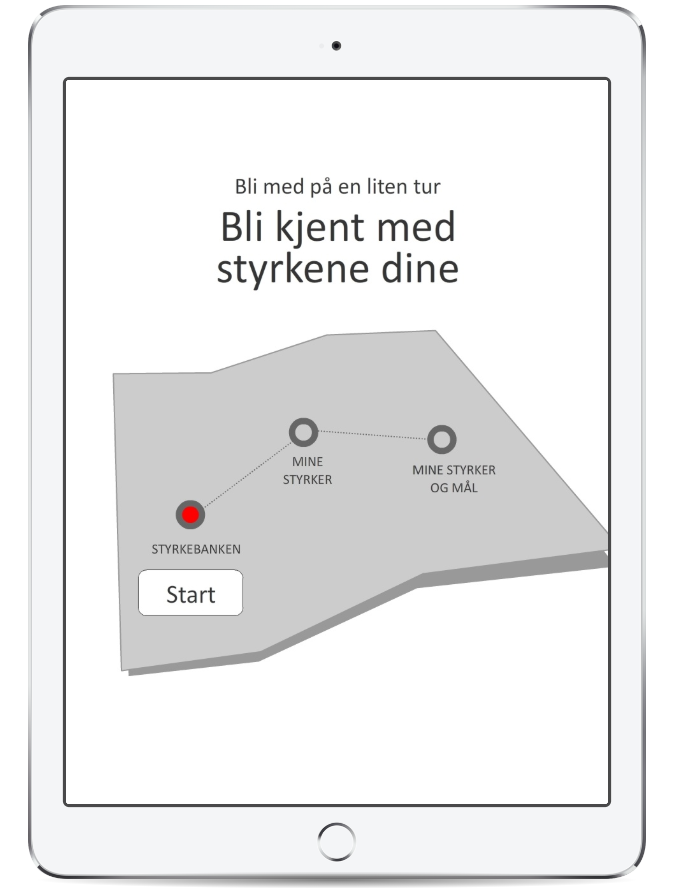


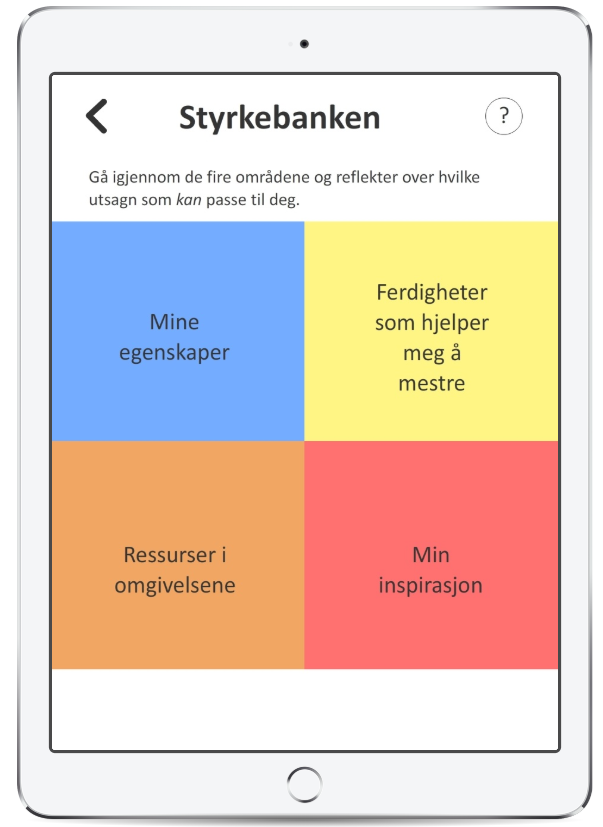


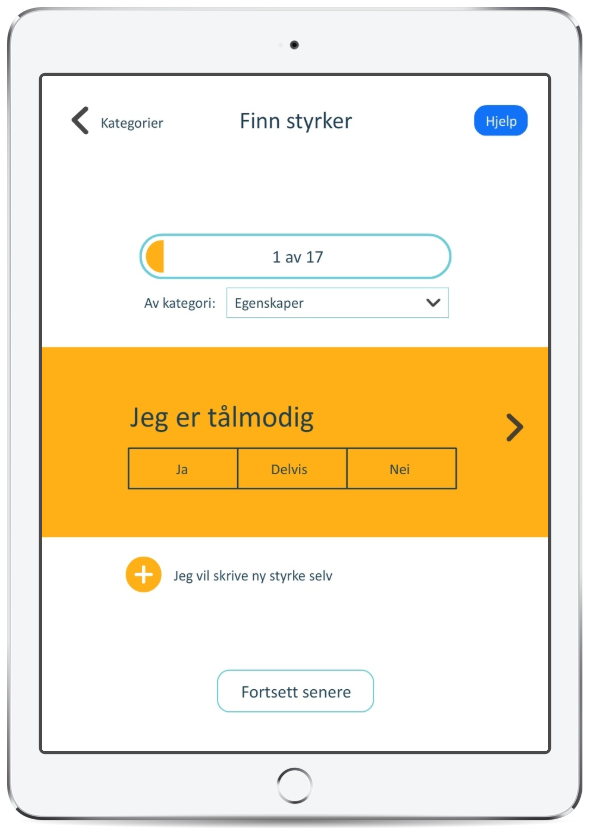


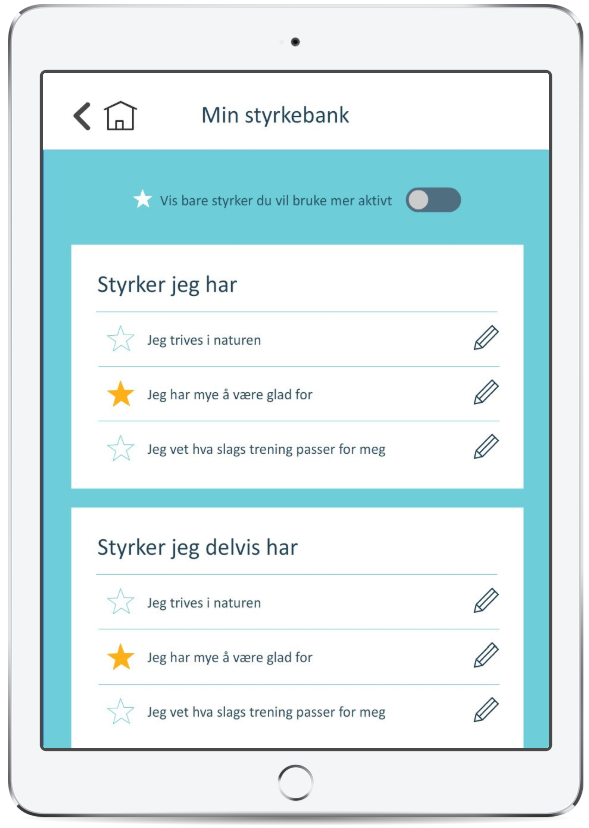


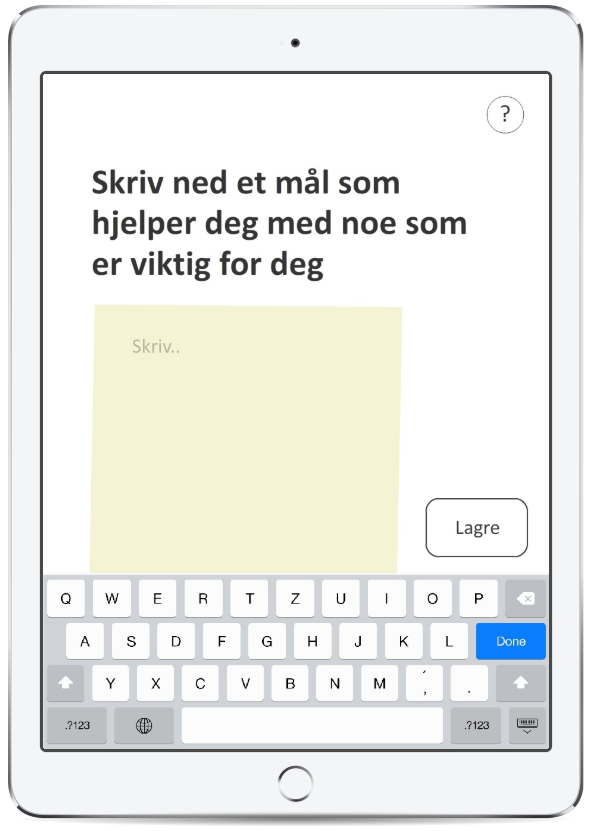

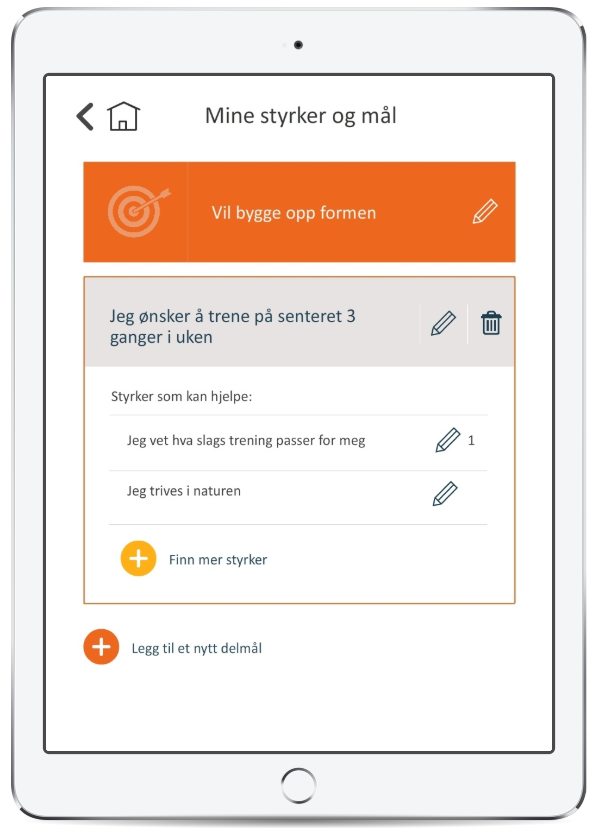

Supplement: Multimedia Appendix 1 [file formative_v4i3e16831_app1.docx]
