# Supplementary material for: Mobile App to Help People With Chronic Illness Reflect on Their Strengths: Formative Evaluation and Usability Testing
Source: JMIR Form Res. 2020 Mar 4;4(3):e16831. doi: 10.2196/16831 (PMC7081135; doi:10.2196/16831)
Supplement: Multimedia Appendix 2 [file formative_v4i3e16831_app2.docx]

Multimedia Appendix 2:

List of strengths used in the Styrkefunn app in Norwegian and English

|  | **Egenskaper** | **Qualities** |
| --- | --- | --- |
| 1 | Jeg er utholdende | I am persistent |
| 2 | Jeg har kunnskap om min sykdom og behandling | I have knowledge about my disease and treatment |
| 3 | Jeg prøver å hjelpe andre | I try to help others |
| 4 | Jeg kan dele mine tanker og erfaringer med andre | I can share my thoughts and experiences with others |
| 5 | Jeg liker å prøve ut eller lære nye ting | I like trying out or learning new things |
| 6 | Jeg kan sette grenser | I can set boundaries |
| 7 | Jeg prøver å være snill mot meg selv | I try to be kind to myself |
| 8 | Jeg er empatisk | I am empathetic |
| 9 | Jeg vet hvilke treningsformer passer meg | I know which types of physical activity suit me |
| 10 | Jeg er stort sett forsonet med min situasjon | I have essentially accepted my situation |
| 11 | Jeg klarer å legge ting bak meg | I can let go of things and move on |
| 12 | Jeg har klart vanskeligheter før og tror jeg klarer det igjen | I have handled adversity before and believe I can do it again |
| 13 | Jeg føler jeg har en viss kontroll i livet mitt | I feel I have some control over my life |
| 14 | Jeg vet at kropp og sinn påvirker hverandre | I know that mind and body influence each other |
| 15 | Jeg tørr å vise sårbarhet | I am not afraid to show vulnerability |
| 16 | Jeg er kreativ | I am creative |
|  | **Glede og mening** | **Joy and meaning** |
| 17 | Jeg prioriterer hva som er viktig for meg | I prioritize what is important to me |
| 18 | Jeg har meningsfylte aktiviteter i hverdagen | I have meaningful activities in my everyday life |
| 19 | Jeg har aktiviteter som gir meg glede og jeg kan se frem til | I have activities that bring me joy and that I can look forward to |
| 20 | Jeg har en positiv innstilling til livet | I have a positive outlook on life |
| 21 | Jeg er ofte i godt humør | I am often in a good mood |
| 22 | Jeg er takknemlig for det gode i livet mitt | I am grateful for my blessings |
| 23 | Jeg har drømmer og håp for framtida | I have hopes and dreams for the future |
| 24 | Jeg har glede av å være i naturen | I enjoy being out in nature |
| 25 | Jeg føler meg verdsatt av andre | I feel valued by others |
| 26 | Jeg har en livstro eller en spirituell praksis | I have a faith or a spiritual practice |
| 27 | Jeg bruker mye humor | I use humor a lot |
|  | **Ressurser i omgivelsene** | **External resources** |
| 28 | Jeg har noen som forstår meg | I have someone who understands me |
| 29 | Jeg har noen som er der for meg hvis jeg trenger det | I have someone who will be there for me if I need it |
| 30 | Jeg har helsepersonell jeg har tillit til | I trust my healthcare providers |
| 31 | Jeg bor i trygge omgivelser | I live in safe surroundings |
| 32 | Økonomisk er jeg i stand til å gjøre ting som er viktig for meg | My economy allows me to do things that are important to me |
| 33 | Jeg har noen som motiverer meg til å leve sunt | I have someone who encourages me to live a healthy life |
| 34 | Mine omgivelser gir meg muligheter til sunn livsstil | My surroundings provide possibilities for a healthy lifestyle |
|  | **Strategier** | **Strategies** |
| 35 | Jeg søker kunnskapen jeg trenger | I seek out the knowledge I need |
| 36 | Jeg søker hjelp når jeg trenger det | I seek help when I need it |
| 37 | Jeg har stort sett en sunn livsstil og tar vare på meg selv | I have a generally healthy lifestyle and take good care of myself |
| 38 | Jeg vet hvordan jeg kan håndtere symptomer | I know how to manage symptoms |
| 39 | Jeg vet hvordan jeg kan håndtere stress | I know how to manage stress |
| 40 | Jeg klarer å sette mål og jobbe mot dem | I am able to set goals and work to achieve them |
| 41 | Jeg balanserer aktivitet ut i fra dagsform | I adjust my activities based on how I feel that day |
| 42 | Jeg lærer av andre som har lignende erfaringer som meg | I learn from others who have been in similar situations |
